# Donation after cardiocirculatory death: a call for a moratorium pending full public disclosure and fully informed consent

**DOI:** 10.1186/1747-5341-6-17

**Published:** 2011-12-29

**Authors:** Ari R Joffe, Joe Carcillo, Natalie Anton, Allan deCaen, Yong Y Han, Michael J Bell, Frank A Maffei, John Sullivan, James Thomas, Gonzalo Garcia-Guerra

**Affiliations:** 1 Department of Pediatrics, University of Alberta, Stollery Children's Hospital; Edmonton Clinic Health Academy 11405-87 Avenue, Edmonton, Alberta, T6G 1C9, Canada; 2 John Dossetor Health Ethics Center, University of Alberta, Edmonton, Alberta, Canada; 3 Department of Pediatrics and Critical Care Medicine, University of Pittsburgh School of Medicine and Children's Hospital of Pittsburgh, 400 45th Street, Pittsburgh, PA, 15201, USA; 4 Department of Pediatrics & Communicable Diseases, University of Michigan Medical School, 1500 East Medical Center Drive, Ann Arbor, MI, 48109, USA; 5 Department of Pediatrics, Pediatric Critical Care Medicine, Janet Weis Children's Hospital, Geisinger Medical Center, 100 N. Academy Ave, Danville, PA, 17822, USA; 6 Golisano Children's Hospital at Strong, University of Rochester School of Medicine, 601 Elmwood Avenue, Rochester, NY 15642, USA; 7 Department of Pediatrics, University of Texas, Southwestern Medical Center; 5323 Harry Hines Blvd, Dallas, Texas, 75390-9063, USA

**Keywords:** Dead donor rule, Death, Donation after cardiac death, Organ donation

## Abstract

Many believe that the ethical problems of donation after cardiocirculatory death (DCD) have been "worked out" and that it is unclear why DCD should be resisted. In this paper we will argue that DCD donors may not yet be dead, and therefore that organ donation during DCD may violate the dead donor rule. We first present a description of the process of DCD and the standard ethical rationale for the practice. We then present our concerns with DCD, including the following: irreversibility of absent circulation has not occurred and the many attempts to claim it has have all failed; conflicts of interest at all steps in the DCD process, including the decision to withdraw life support before DCD, are simply unavoidable; potentially harmful premortem interventions to preserve organ utility are not justifiable, even with the help of the principle of double effect; claims that DCD conforms with the intent of the law and current accepted medical standards are misleading and inaccurate; and consensus statements by respected medical groups do not change these arguments due to their low quality including being plagued by conflict of interest. Moreover, some arguments in favor of DCD, while likely true, are "straw-man arguments," such as the great benefit of organ donation. The truth is that honesty and trustworthiness require that we face these problems instead of avoiding them. We believe that DCD is not ethically allowable because it abandons the dead donor rule, has unavoidable conflicts of interests, and implements premortem interventions which can hasten death. These important points have not been, but need to be fully disclosed to the public and incorporated into fully informed consent. These are tall orders, and require open public debate. Until this debate occurs, we call for a moratorium on the practice of DCD.

## Introduction

There have been many "consensus" statements addressing the practice of donation after cardiocirculatory death (DCD). In general, these claim that DCD conforms to clear ethical principles, respects the dead donor rule, and is worthy of support [1-5]. Many believe that the ethical problems of DCD have been "worked out" and that it is unclear why DCD should be resisted. The dead donor rule is an "unwritten, uncodified standard that has guided organ procurement in the United States since the late 1960s" [6]. This rule claims that humans must be dead before vital organs can be taken, and is intended to prevent the following: patients being killed by organ retrieval, harm or exploitation of the weak/vulnerable, mistrust of doctors and transplantation, and treating a patient merely as a means to organs [6,7].

We argue for a moratorium on the practice of DCD until full public disclosure and fully informed consent is obtained from potential donors. Specifically, we will argue that DCD donors may not yet be dead, conflicts of interest in the decision to withdraw life support before DCD are unavoidable, potentially harmful premortem interventions during DCD cannot be justified even with the rule of double effect, consensus statements are of low quality and plagued by conflict of interest, and that claims that DCD conforms with the intent of the law and current accepted medical standards are misleading and inaccurate. Moreover, some arguments in favor of DCD, while likely true, are "straw-man arguments," such as the substantial benefit of organ donation.

## I The Process of DCD

In general, the current practice of controlled DCD can be summarized as follows [1-5]. First, there is a decision based on the patient's wishes (either directly, or via a substitute decision maker) or best interests (via a guardian surrogate decision maker when the patient's wishes either are not known, or the patient was never competent) to discontinue life support therapy. This is typically made in the situation of severe brain, neuromuscular, or organ dysfunction when the burdens of continued life support are felt to outweigh the benefits of delaying death. Second, the patient/surrogate/guardian is offered the "opportunity" for organ donation after death if indeed death is pronounced by irreversible lack of circulation. Third, after consent, the patient is withdrawn from life support and death is awaited. If the circulation stops within 1-2 hours, the patient is a DCD donor. Fourth, death is declared after 2-10 minutes of absent circulation, the time varying between hospitals and countries, with the mechanism to determine absent circulation varying as well. Fifth, the surgical team, at the 2-10 minute mark, begins surgical harvest of the organs appropriate for donation. Often cannulas will have been inserted in the femoral vessels prior to life support withdrawal to facilitate rapid organ preservation at the 2-10 minute mark. Usually medications such as heparin and phentolamine will have been given to the patient prior to absent circulation to theoretically improve organ preservation. This orchestrated expected death is called "controlled DCD" to contrast with "uncontrolled DCD" which refers to donation after unexpected cardiac arrest with death pronounced after failed attempts at cardiopulmonary resuscitation.

## II The Consensus Position

Protocols for DCD have attempted to clarify the justification for this practice with a series of self-evident truths [1-5]. First, the decision to withdraw life support is made before any mention of DCD, and is not influenced by the option of DCD. Indeed, the physicians who discuss withdrawal are not in any way involved in transplantation. Second, fully informed consent for DCD is freely obtained for organ donation after death and for any interventions done premortem. Third, after 2-10 minutes of absent circulation this state is permanent. There are no cases of circulation restarting on its own after this time period, and to try to restart circulation by cardiopulmonary resuscitation is unethical given the patient's wishes to have a do not resuscitate order. For these reasons, the absent circulation is irreversible, and satisfies the legal, ontologic, and common-sense requirement of irreversibility of death. Fourth, premortem interventions are done solely with the intent to improve donated organ function, and if there is any potential to hasten or cause death this effect is both unintended and unavoidable. Finally, declaring death based on permanent absence of circulation at 2-10 minutes conforms with accepted medical standards, and with the intent of the law regarding irreversibility and criteria for death. We will argue that none of these claims can withstand careful scrutiny.

## III The Irreversibility of Death

In discussing death it is useful to review the paradigm used to define it: death is an irreversible biological/ontological event. As Bernat has argued, death is an event separating the process of dying (living, while it seems death is near) from the process of disintegration [8-10]. Bernat argues that death is a biological univocal ontologic state of an organism, and irreversible ("if the event of death were reversible it would not be death but rather part of the process of dying that interrupted and reversed" [[8]p37]; "no mortal can return from being dead, any resuscitation or recovery must have been from a state of dying ") [[8,9]p124,8]. Others have argued for the same paradigm, including the President's Commission [11]. Philosophically, death is the irreversible state where there has been loss of the integrative unity of the organism as a whole; the organism is no longer more than the sum of its parts, and irreversibly cannot resist the disintegration entailed by the forces of entropy [9-12]. The problem is that this accepted conception of death and irreversibility is not compatible with DCD. We aim to clarify the debate in the literature on this point (Table 1).

**Table 1:** Clarification of the arguments surrounding the interpretation of the 'irreversibility' of death.

Absent circulation is irreversible at 2-10 minutes	Absent circulation is not irreversible at 2-10 minutes
Permanent is a reasonable 'construal' of irreversible.	The ordinary meaning of irreversible is 'not capable of being reversed.' Permanent is not a 'construal' of irreversible at all.

There is a moral/legal obligation not to resuscitate.	Irreversible is not a moral/legal concept. The obligation to or not to resuscitate is due to the patient being alive. Death is a state of a body, and those in exact states cannot be both dead and alive.

There is no difference in outcome by waiting for irreversibility.	This admits that permanence is a prognosis of death, not a diagnosis of death. The DCD donor is living (even if he/she may be dying).

Autoresuscitation does not occur after 65 seconds of absent circulation.	This is based on inadequate data (n = 5), and tries to explain away the Lazarus phenomenon.

Permanence accords with accepted medical standards and the intent of the law.	This is misleading and inaccurate. This ignores ontologic and moral issues. This mischaracterizes accepted medical standards. The intent of the law was not 'permanence'.

Brain death is not required to diagnose death.	The intent of the law is that there is only one death per person. DCD donors are not brain dead.

### Ontology and 'construals' of irreversibility

The ordinary sense of the meaning of irreversible is "not capable of being reversed," [13] and it "depends on what physically can or cannot be done" [[14]p77]. The plain meaning is that "no known intervention could have eliminated it" [[15]p26]. If a condition is never actually reversed it is permanent, but if a condition never could be reversed it is irreversible [15]. In other words, irreversibility entails permanence; permanence does not entail irreversibility [15]. The consensus on the moral acceptability of DCD argues that a so-called weak 'construal' of 'irreversible' is 'permanent' [1-5]. We argue that 'permanent' is not a construal of 'irreversible' at all; indeed, Bernat agrees when he writes "the weakest construal falls outside the domain of irreversibility altogether, and resides properly within the domain of permanence" [[9]p125]. Marquis provides an example that makes this commonsense point:

Suppose that Joe has a heart attack and his circulatory function stops. Fred, a physician standing next to Joe, refuses to perform CPR on Joe because Joe is a rival... Suppose that CPR would have been successful, but because it was not performed, cessation of Joe's circulatory function was permanent. Was Fred's refusal to act wrong? Not if we understand the irreversible cessation of circulatory function as equivalent to the permanent cessation of circulatory function... On that understanding, Joe was dead as soon as he collapsed, and Fred's failure to perform resuscitation was not wrong, for he had no obligation to resuscitate a corpse [[15]p26].

### Moral decisions and the ethical/legal obligation not to resuscitate

An objection to this scenario would be that in DCD an informed autonomous moral decision has been made not to attempt resuscitation, and this is why permanent is an acceptable 'construal' of 'irreversible' in determining death [1-5]. This initially persuasive argument fails. As Alister Browne writes, this tries "to perform the trick of explaining how a physically reversible state can be described as 'irreversible' by treating irreversibility as a legal/moral concept... In ordinary usage, whether a person is dead or alive depends on what physically can or cannot be done to reverse that state, not on what legally/morally can or cannot be done" [[14]p77]. Marquis elaborates on three problems with appealing to this normative ethical sense of 'irreversible' [15]. First, 'reversible' is a dispositional property having the capacity to exhibit the occurrent property of 'being reversed' in the appropriate circumstances. Similar dispositional properties are terms like 'fragile', and 'soluble'. So, the question is, does it make sense to say: it would be wrong (ethically) for me to deliberately break your fine china, therefore, your fine china is not fragile; or it would be wrong (ethically) for me to dissolve your gold ring in acid, therefore, your ring is insoluble. Second, the necessary condition of having an obligation to not resuscitate the DCD donor is that the donor patient is alive. In the example above, Fred had an obligation to resuscitate Joe because a "necessary condition of Fred's having an obligation to Joe is that both Fred and Joe exist [are alive]" [[15]p28]; that is why it was wrong for Fred not to resuscitate Joe. Similarly, in DCD, it would be wrong for a physician to attempt to resuscitate the donor patient. But, this is because death's irreversibility has ethical consequences, the fact that a person is dead is the basis for a change in our obligations, and the donor patient was not yet dead. Third, many patients in exactly the same state would be considered dead or alive depending on whether resuscitation will be attempted; "but death is a state of a body" [[15]p29]. Truog makes this point when he describes three patients in the identical physiological state who are dead (the DCD donor), alive (CPR is started), and alive (the DCD donor whose parents change their minds and demand resuscitation at 5 minutes) [16]. Marquis offers another example:

An individual is in a severe automobile accident and arrives in the ER. You are the ER physician. You judge that the patient's blood loss is so great that the patient will soon die unless she receives a blood transfusion. Her surrogates decline the transfusion because she is a Jehovah's Witness. You respect the refusal and she dies. You would say, of course: 'Her condition was reversible! I wish I could have transfused her!'... you would be wrong to say that... since reversing the patient's condition was not legally or morally permissible, the patient should have been viewed as being in an irreversible condition...[[15]p29].

Although some may argue that the state is just as good as death, or is very close to death, we cannot say it *is *death. "To say it is makes a word mean what it does not, and to do that without warning is necessarily to mislead" [[14]p78].

### Consequentialism, utility, and prognosis

It is interesting that the proponents of DCD may in some way recognize these flaws in the declaration of death in the donor. Consensus statements simply state that 'permanent' was the 'construal' of 'irreversible' accepted by the panels [1-5]. More recently, it has been admitted that the declaration of death in the donor is "a compromise on biological reality" [[9]p128], "an approximation" [[8]p41], "inconsequential" [[9]p129], "a valid proxy... produces no difference in donor outcome" [[5]p975], and a perfect indicator/surrogate/proxy of irreversible [8-10]. In other words, "permanent cessation of circulation constitutes a valid proxy for its irreversible cessation because it quickly and inevitably becomes irreversible and because there is no difference in outcome between using a permanent or irreversible standard" [[17]p14]. Indeed, it has been argued on a utilitarian basis that, "the good accruing to the organ recipient, the donor patient, and the donor family resulting from organ donation justified overlooking the biological shortcoming because although the difference in the death criteria was real, it was inconsequential' [[8]p41].

We believe that these arguments may be used to make a case for organ donation in the setting of DCD; however, they cannot be used to argue that the donor is dead. What these arguments show is that the DCD donor has an almost certain prognosis of death, is in the process of dying, but is still living and not yet dead. Moreover, the accuracy of the prognosis depends on a future event (whether resuscitation will be attempted, whether autoresuscitation will occur), and to claim the prognosis is certain relies on backward causation. In philosophical terms, the prognosis is a "soft fact" about the past, not determined until the future events occur, and thus not a "hard fact" at all. It is a "serious logical mistake by conflating a prognosis of imminent death with a diagnosis of death" [[16]p16]. If certain prognosis of death was equivalent to death, then certain patients that are clearly alive would have to be classified as dead; Truog mentions the quadriplegic ventilator dependent man having ventilator withdrawal, the patient dependent on ECMO due to no underlying cardiac function having withdrawal of ECMO, and the patient dependent on tube feeds having these withdrawn [18]. Is a drowning man dead because no one will swim out to save him? Or is he merely going to die? It is a separate question whether the patient can still be a donor while violating the dead donor rule. There is also the question of whether the donor's prognosis is certain.

### Autoresuscitation and the Lazarus phenomenon

The consensus statements that approve of DCD were of poor quality when it comes to evidence based medicine, and based on "expert opinion" [1-5]. It is repeatedly claimed that there has been no case of autoresuscitation occurring more than 65 seconds after loss of circulation [1-5]. It is important to point out that this crucial 'fact' is based on very poor data limited by: small numbers (n = 108) in 5 studies conducted from 1912-1970 of patients aged 9 months to 87 years, poorly described patient selection criteria, no description of whether there was continuous clinical monitoring other than ECG, no arterial line monitoring, many patients who did have resuscitation attempts, and only 5 cases where ECG monitoring was stated to have continued more than 2 minutes after loss of cardiac activity (Table 2) [19-23]. Remarkably, one of these studies reported a 25 year old woman who had asystole for 2.5 minutes followed spontaneously by an atrioventricular rate of 33/min [21].

**Table 2:** Case series claimed to document lack of autoresuscitation.

Study (year)	n	Selection criteria	Monitoring > 2 min*	AL	Cont obs	Author's conclusions	Comment
Stroud et al (1947) [19]	23	"When practical, the electrodes were attached to the moribund patient before clinical death... tracings were taken intermittently or continuously until the string became motionless." Age 10-87 yr; 1 child age 10 yr.	stated for n = 2 (adults)	0	not stated	"Permanent standstill occurred without VF in 50% of cases."	n = 2 not monitored at death, leaving a true n = 21.
Enselberg (1952) [20]	43	"EKGs were recorded for varying lengths of time before, during, and after death... resuscitative measures were applied in 22 cases." Age 8-80 yr; 2 children ages 8 yr and 14 yr.	not stated	0	not stated	"Recurring asystoles or ventricular standstills are common and often appear to be self-limited."	"It is possible that in many cases the recording of terminal EKGs may have been stopped upon the appearance of a long asystole, before true cessation."
Robinson (1912) [21]	7	"EKG records obtained from 7 patients before and during the actual stoppage of the heart... There were naturally many failures to obtain records, especially when fatalities occurred suddenly." Age 9 mo-37 yr; 3 children ages 9 mo, 18 mo, and 4 yr.	stated for n = 1 (age 9 mo)	0	not stated	"Cardiac activity continued from 6-35 min after all the usual clinical signs of death had occurred."	-Case 5 had resumption of cardiac rhythm at a rate of 33 bpm "after a stoppage of 2 1/2 minutes." -Case 7 had no evidence of cardiac activity "17 min post mortem. Because the records were not satisfactory, a more detailed analysis is not possible."
Willius (1924) [22]	6	"... six patients in whom almost continuous EKG records were obtained from 10 min to 7 hr 32 min preceding death." Age 29-58 yr (based on information for n = 4).	not stated (4), several minutes (1), 1 min 3.04 sec(1).	0	not stated	"The changes occurring in the mechanism of the human heart preceding and during death are variable..."	-
Rodstein et al 1970 [23]	31	"A series of aged individuals in whom terminal EKGs and necropsies were available... Lead II was then continuously recorded until electrical activity ceased... The time of cessation of electrical activity- electrical death- was recorded. Where clinically indicated, the usual resuscitative measures were employed." Ages 73-101 yr.	stated for n = 1.	0	not stated	"The majority of deaths from all causes showed an EKG pattern of the dying heart..." In review of the literature they report "survival times after clinical death [of up to 50 min]."	"In 7 (23%) of the 31 patients, electrical death terminated in VF... One patient terminated with a rapid VT."
TOTAL	110	Selection criteria poorly described.	n = 5	n = 0	not stated	variable terminal EKG patterns	cannot determine if autoresuscitation occurs

AL: arterial line; EKG: electrocardiogram; VF: ventricular fibrillation. Cont obs: continuous observation. *Monitoring > 2 min: refers to ongoing EKG monitoring for > 2 min after death was pronounced based on EKG asystole or VF. This Table is modified and reproduced with permission of the author, and was originally published in [37].

Another form of autoresuscitation is called the Lazarus phenomenon, which has been reported in at least 32 cases [24]. These published cases describe return of unassisted spontaneous circulation from a few seconds to 33 minutes after failed CPR, although most were not adequately monitored to determine the exact timing of return of circulation [24]. Nevertheless, some cases have had constant EKG monitoring with constant observation and 6 of these had autoresuscitation at 5-7 minutes after absent circulation and asystole; some have had arterial line monitoring in addition to continuous EKG monitoring and constant observation, with 3 of these having autoresuscitation at 3-5 minutes after absent circulation and asystole; and some have had constant observation and were found to have autoresuscitated 8-10 minutes after absent circulation with asystole (Table 3) [25-37]. These cases are hypothesized not to be similar to withdrawal of life support as in the setting of DCD, because they all occurred after failed CPR [24]. The argument is that dynamic lung hyperinflation during CPR may decrease venous return and, after stopping ventilation, this will reverse and autoresuscitation occur; or, that there was delayed delivery of resuscitation drugs to the heart that led to delayed autoresuscitation [24]. The problem is that these do not explain the cases.

**Table 3:** Selected reported cases of autoresuscitation.

Author	Age (yr)	Diagnosis	Rhythm	Min*	Outcome	EKG	AL	Obs
Letellier [25]	80	Pulmonary edema	asystole	5	normal	+	-	+
Voekkel [26]	55	Sudden death	asystole	7	death at 3 d	+	-	+
MacGillivray [27]	76	COPD	asystole	5	death 24 hr later	+	-	+
Rosengarten [28]	36	asthma	EMD	5	normal	+	-	+
Abdullah [29]	93	sepsis	asystole	5	not stated	+	?	+
Al-Ansari [30]	63	COPD	asystole	3	normal	+	-	+
Frolich [31]	67	MI	asystole	5	normal d3; death d7	+	+	+
Casielles-Garcia [32]	94	hemorrhage	EMD	3	death at 18 d	+	+	+
Maleck [33]	80	sepsis	asystole	< 5	death at d2	+	+	+
Quick [34]	70	hyperkalemia	asystole	8	normal	-	-	+
Ben-David [35]	66	sudden VF	asystole	10	normal	-	-	+
Monticelli [36]	78	MI	asystole	> 10	death at 19 hr	-	-	+

Monitoring of the patient is either present (+) or absent (-); the mode of monitoring is by continuous electrocardiogram (ECG), arterial line (AL), and/or continuous clinical bedside observation (Obs). COPD: chronic obstructive pulmonary disease; EMD: electromechanical dissociation; MI: myocardial infarction; VF: ventricular fibrillation. *Min: time in minutes from stopping resuscitation in the stated rhythm to return of circulation.

This Table is modified and reproduced with permission of the author, and was originally published in [37].

There are likely two types of Lazarus phenomenon: one occurs after a short interval of 1-2 minutes and may be due to resolution of hyperinflation with associated venous return and drug delivery to the heart; the other type with more delayed autoresuscitation cannot be so explained [37,38]. Hyperinflation resolves within seconds of stopping ventilation, as shown by studies documenting lung derecruitment within seconds of disconnection from the ventilator; drug delivery to the heart during asystole is difficult to explain [39]. It is more likely that the Lazarus phenomenon is underreported, and that after CPR (with monitoring more likely to occur) it is more likely to be detected. The Lazarus phenomenon indicates that autoresuscitation can occur after absent circulation of many minutes.

### Accepted medical standards and the intent of the law

Prominent DCD proponents have claimed that determining death is not primarily an ontologic issue (whether the biological state of the organism is dead), nor a moral issue (whether our obligations to the patient are as if they are dead); rather, it is "fundamentally a medical practice issue" [[17,40]p1762]. Although ignoring ontology and moral issues is at best concerning, we will examine the claim nevertheless. There are two components to this claim. First, when physicians declare death based on cardiocirculatory criteria in non-DCD settings, they declare death at the moment they verify loss of circulation, without a waiting period [2,5]. Second, the intent of the law, the President's Commission in Defining Death, and the Uniform Determination of Death Act, was a permanence standard of loss of circulation, in accordance with the accepted medical standards [4,5]. Both of these claims are misleading, and inaccurate.

First, the observation that physicians often do not have a waiting time to verify irreversibility of absent circulation when commonly declaring death "is irrelevant in situations where following the dead donor rule (DDR) requires knowing whether the patient is merely dying or already dead... the accuracy that is required of our assessments depends on the consequences of our assessments being wrong... if one holds that the DDR is an inviolate principle of organ donation, then the difference between 'dying' and 'dead' becomes crucial" [[16]p16]. Joffe argued:

[the lead author of the recent Canadian forum on DCD] writes that in the past "observation and confirmation was not required and the irreversibility of death was not a practical concern, although diagnostic errors were made." Acknowledging that "diagnostic errors were made" shows that what death is has been clear in the past. Outside the context of organ donation, when one was claimed to be dead based on the irreversible loss of circulation, if the patient was subsequently revived (by autoresuscitation or intervention) and clearly alive, one simply had to admit that the pronouncement of death was incorrect. I do not believe that in this situation one would continue to insist that the diagnosis of death was correct, and that somehow the patient was revived from the irreversible state known as death. This shows that death in the past was understood as an irreversible state of dis-integration of the organism. However, in the context of DCD, we are now forced to "enhance the rigour of the determination of death," and would in this situation of revival have to explain somehow that the patient in the irreversible (or "permanent") state 'death' has now somehow become alive [[37]p4].

Accepted medical practice is to declare death when circulation is lost, and retrospectively know this was correct after a period of time that verifies it is irreversible. If this retrospective ability is taken away, then we believe physicians would be aware that death had not yet occurred when circulation stopped.

Second, the claim that the intent of the law, the President's Commission on Defining Death, and the UDDA was a permanence interpretation of irreversible is not tenable. The opinion that permanence *was *the intent seems to have evolved over time, with Bernat in earlier writings suggesting that it "*may*" have been the intent, and even earlier, that it was clearly *not *the intent [8,9]. In the late 1990s both Bernat and Capron (the Executive Director of the President's Commission) clearly did not think it was the intent. Capron wrote in 1999:

The Pittsburgh protocol seems less a challenge to the UDDA than simply a contradiction of it. The failure to attempt to restore circulatory and respiratory functions in these patients prevents lawfully declaring that death has occurred because irreversibility must mean more than simply 'we choose not to reverse, although we might have succeeded'... the actual point in each case at which it becomes impossible to reverse the loss of functions would be unaffected... in other words, 'It's hopeless'- he would be confusing a prognosis for a diagnosis... Thus, replacing 'irreversible cessation of circulatory and respiratory functions' with 'we choose not to reverse' flies in the face of the UDDA's underlying premise [[41]p132].

Bernat wrote in 1998:

The cessation of heartbeat and breathing must be prolonged because their absence must be of sufficient duration for the brain to become diffusely infarcted and for the cessation of heartbeat and breathing conclusively to be irreversible... It takes considerably longer than a few minutes for the brain and other organs to be destroyed from cessation of circulation and lack of oxygen. Moreover, it takes longer than this time for the cessation of heartbeat and breathing to be unequivocally irreversible, a prerequisite for death. As proof of this assertion, if cardiopulmonary resuscitation were performed within a few minutes of cardiorespiratory arrest, it is likely that some of the purportedly 'dead' patients could be successfully resuscitated to spontaneous heartbeat and some intact brain function... The brief absence of heartbeat and breathing is highly predictive of death in this context [of DCD], but that at the time the organs are being procured in the Pittsburgh protocol, death has not yet occurred [p20]... the UDDA is a useful statute that has been implemented in the majority of states in the United States. Because of its unambiguous wording, succinctness, and for the sake of uniformity, I continue to support its adoption...[[42]p21].

With these writings in mind, it is remarkable the change that has occurred in more recent writings. For example, Bernat wrote that "[the 2006] national conference affirmed the ethical propriety of DCD as not violating the dead donor rule" [[3]p281]. Bernat and Capron wrote that although permanent cessation of circulation has occurred before irreversible cessation of circulation, "it is ethically and legally appropriate to procure organs [because this]... is the meaning of 'irreversible' in the Uniform Determination of Death Act" [[5]p963]. Bernat also wrote that "permanent was the intended construal of irreversible in the UDDA" quoting personal communication from Capron Dec 15, 2008 [[17]p15], and March 17, 2009 [10]. Other authors who were part of the President's Commission on Defining Death have also expressed concerns with DCD protocols, suggesting the intent of the law was not a permanence standard [43-45]. The legislation in some states, and the case law from the few cases that exist also make it clear that permanent is not an acceptable interpretation of irreversible [46]. Some have argued that DCD actually is at high risk of breaking the law [47]. It has also been pointed out that "wherever the Commission used the word 'permanent', it was followed by a description of loss of function that cannot recover because of ischemia, damage, destruction, or necrosis [i.e. irreversible damage]. Neither intent nor action not to resuscitate was mentioned as contingencies qualifying 'permanent' as 'irreversible'" [[48]p1498,49]. Rady et al also ask "did the President's Commission intend for 'irreversible' to have different meanings within the uniform determination of death act when determining death by a circulatory standard vs. a neurologic standard?" [[48]p1498,49] In brain death it is accepted that the lost functions must be irreversible, not merely permanent. In DCD, it apparently is not.

### Brain death: two deaths, or one death?

Medicine, law, ethics, the President's Commission in Defining Death, the Law Reform Commission in Canada, and proponents of DCD all agree that there is only one death per person [11,41,42,50]. There is one phenomenon, or state, of death. There happen to be two ways to diagnose this unitary state: the tests to confirm the irreversible loss of all critical clinical functions of the brain including the brainstem, and the tests to confirm the irreversible loss of cardiocirculatory and respiratory functions. The state of brain death, so the argument goes, is the state of death, and irreversibly absent cardiocirculatory functions is just the usual way of determining this state of the brain [11,12,51]. As Capron explains, "The reason for alternative standards for determining death is not that we believe there are two kinds of death. On the contrary, there is one phenomenon that can be viewed through two windows, and the requirement of irreversibility ensures that what is seen through both is the same or virtually the same thing. Disregarding the requisite of irreversibility as it applies to either standard is as destructive to the process of determining death as it would be to ignore the requisite of cessation" [[41]p133]. This well accepted state of affairs is currently being ignored in the setting of DCD.

At 10 minutes of absent circulation the brain is not destroyed, and if resuscitation were implemented, some will survive with some clinical and critical brain functions [52-57]. It is very likely that over 15 minutes of absent circulation is required before one can claim that brain death will be likely [11,44,52-57]. Proponents of DCD have argued that permanent absence of circulation is a perfect surrogate for inevitable brain destruction, again conflating prognosis with diagnosis [5]. They also argue that the law states that death can be determined by brain death tests or circulatory tests, not both, ignoring the clear intent of the law [4,5]. They also point out that after less than a minute of absent circulation some studies show there is loss of electroencephalographic brain activity, confirming lack of critical clinical brain functions [4,5]. This argument is odd at best. Electroencephalographic activity detects only cortical activity, and for this reason has been questioned as an ancillary test to diagnose brain death; it does not rule out subcortical activity, nor critical brainstem functions [58]. If one was serious about being sure clinical brain death was present, relying solely on an EEG would be dangerous. Moreover, a recent study of seven adults having withdrawal of life support found all had surges of electroencephalographic activity when the patients were pulseless on arterial line, motionless, and with asystole or ventricular escape rhythms; these surges "last for a few minutes at maximum, but usually last between 30-180 seconds" [59].

### At the bottom of (or beyond) the slippery slope

We believe that not engaging the arguments we have made questioning some aspects of DCD has led to serious problems. Although an appeal to a utilitarian judgment is tempting, given the good consequences (beneficience) from organ donation, we believe there are also negative consequences. Alister Browne points to some "good-looking" moral principles being violated in DCD: never treat others as mere means, never interfere with the liberty of individuals when they are not doing or threatening harm to others, and never keep information concerning matters of public policy from the people in a democracy [14]. He also points out some undesirable effects: the consequences of discovery of the deception, whether it will set a precedent for deception elsewhere, how it will affect the character of those engaged in it, and the effect on democratic procedures and institutions [14]. He urges that we "see the issues clearly and face them squarely, to understand the choices for what they are, and ourselves for who we are" [[14]p85].

For proponents of DCD, the reason permanent is a surrogate for irreversible is that the team and patient/surrogate have decided that attempts to reverse it (which we know would usually be successful) will not be allowed ethically/legally to occur because there is an agreed upon DNR order in place. In the setting of uncontrolled DCD this has resulted in the reinstitution of CPR (manual or by machine), or even institution of cardiopulmonary bypass (extracorporeal life support), once death is declared, in order to preserve the organs [60-63]. So, the result is a full circle: the patient is dead because we can use a weak 'construal' of 'irreversible', and once that is accepted and the patient is declared dead, we can resuscitate them with CPR and extracorporeal life support, the exact things that were forgone to allow 'death', that were claimed legally disallowed and to justify the creative definition of 'irreversible'.

There are other reasons why our concerns with DCD are even greater in this setting of uncontrolled DCD (Table 4). It is noteworthy that in patients who are dying with absent circulation that have CPR often for over 1 hour, institution of extracorporeal life support (Extracorporeal-CPR) is associated with good survival and neurological outcome in over 40% of adults and children [64-74]. We agree that "the thin line between life and death, between rescue ECLS and in situ organ perfusion" has been crossed [[74]p753]. The Institute of Medicine wrote that this practice should be supported [75-79]. To be fair, a recent consensus statement admitted that this practice "retroactively negates" the death diagnosis, and should not be done [5]. It is hard to see how an irreversible state can be retroactively reversed. A member of the President's Commission warned of this in advance:

**Table 4:** Increased concerns with the practice of uncontrolled donation after cardiocirculatory death.

Area of concern	Examples
The decision to withdraw life support is independent of the DCD decision.	The decision to stop CPR is not independent of organ donation. As soon as CPR is stopped, it is clear that organ donation procedures will start. The decision to stop CPR is therefore a decision whether to attempt to save the life versus identify the patient as a donor.
Informed consent is obtained for DCD.	Consent is not truly informed. First, a signed donor card is a legally binding and irrevocable decision, but unlikely informed [78,160]. Second, organ preservation is started based on an "opting-out" system, prior to determination of donor status and prior to contacting the family [79]. This "protects rather than infringes the family's prerogative to make decisions [about organ donation]" and "enhances autonomy", allows the family the "opportunity to donate", "preserves family choice", and is an "expression of respect" for the family's choice [75-77]. This assumes that the surgical steps taken to preserve organs are "modest", "minimally invasive", and "only slight" [75-79]. These are at best arguable claims.
Absent circulation for 2-10 minutes is permanent, and therefore is diagnostic of death.	The IOM claims that a "hands off period could be very brief and may even be unnecessary" [75], apparently ignoring the cases of Lazarus phenomenon after stopping failed CPR. In addition, re-starting CPR and/or ECMO clearly reverse the absent circulation, and often allow resumed brain activity, and in the context of ECMO, often allow survival with good neurological outcome [64-74].
Death declaration conforms with accepted medical standards and with the intent of the law.	The accepted medical standard when using ECMO to rescue a patient during failed CPR is to cool the patient for 24 hours, then slowly re-warm, and then assess prognosis cautiously.

Quite often, the carefully wrought initial protocols give way over time to a more 'pragmatic' approach, ultimately allowing interventions that would not have met the stringent initial conditions... Past experience demonstrates that efforts to evaluate the current protocol must anticipate that its current restrictions are likely to be relaxed significantly, here and elsewhere, once the protocol is endorsed in principle and put into practice. In my own view, approving the protocol on the basis that its current restrictive conditions will continue to provide adequate protections is an exercise in self-delusion [[43]p229].

Arnold and Youngner also warned of the "quieter strategy of policy creep" [6].

## IV Conflicts of Interest, the Withdrawal of Life Support Decision, and the DCD Decision

### The decision to withdraw life support cannot be separated from consideration of DCD

Most DCD statements are clear that conflicts of interest shall not influence decisions [1-5]. We believe that this is impossible. First, it is said that the decision to withdraw life support will be independent of the request and decision regarding DCD. The physician discussing withdrawal of life support will be aware of the future option of DCD and will not be able to prevent this from influencing his/her opinion. Knowledge and experience of the great benefit to patients with organ failures from organ transplantation, of several patients in the hospital now or recently with these organ failures who are desperately awaiting an organ, and of the academic and financial prestige to the institution and colleagues from organ transplantation activities are unavoidable. The psychology of decision making is complex, but it is clear that bias need not be consciously intentional, and that unconscious biases are more potent and pervasive [80,81]. In addition, disclosure of conflicts of interest, while morally required, do not improve the situation, and have been shown to worsen the influence of bias on decisions [82]. Further, it will not be possible to ensure public acceptance of DCD without prospective donors themselves being aware of the possibility of DCD when they are critically ill.

Second, the risk of bias due to the availability of DCD cannot be simply acknowledged and stated to be unacceptable, therefore allowing one to pretend it has gone away. A Canadian multicenter study found that of 341 adult patients who were assessed by a physician on at least one occasion to have a probability of ICU survival of < 10%, 99 (29%) survived the ICU [83]. Even for those where this prediction was made on at least three occasions, the actual survival was 27/120 (22.5%). When the physician predicted a chance of survival of < 10%, patients were more likely to have withdrawal of life support, and this prediction more powerfully predicted ICU mortality than illness severity, evolving or resolving organ dysfunction, use of inotropes or vasopressors, age, and prior functional status [83,84]. Other studies have found large variability in the accuracy of prognostication by intensivists [85]; in the thresholds for and rates of withdrawal decisions among intensivists, ICUs, and hospitals [86-92]; and that this can and does lead to self-fulfilling prophesies in predicting outcomes [93]. In the Canadian multicenter study, 3.6% of patients having withdrawal of mechanical ventilation in anticipation of death were discharged home [84]; and in an international ICU adult study the proportion of hospital survivors that had withdrawal/limitation decisions ranged from 2.4-30.3% [92]. The concern that DCD will unduly bias these subjective decisions about withdrawal of life support and alter outcome has been raised by others [94-96]. We suggest that this bias can have major implications for patient prognosis from critical illness.

### The decisions cannot be independent of transplant personnel

Third, proponents of DCD claim that those involved in transplantation will not be the ones who discuss DCD and obtain consent from the patient/surrogate. This is at best misleading. It may be true that the transplant surgeons will not be the ones to explain and request consent for DCD. However, it is not accurate to claim that "no physician who has had any association with a proposed transplant recipient that might influence their judgment shall take part in the determination of death" nor that "attending hospital staff caring for the recipient should be different than staff caring for the donor" [[4]pS9]. The physicians and nurses caring for terminally ill ICU patients, discussing withdrawal of life support, and discussing DCD, are the same ones who care for critically ill potential organ recipients and critically ill postoperative transplanted patients. Whether they care for the exact recipient of their most recent patient's donated organ is irrelevant. They care for both groups of patients and this creates an unavoidable conflict of interest.

Fourth, we believe that conflict of interest matters are actually encouraged in order to improve adoption of DCD. The organ donation breakthrough collaborative (supported by the U.S. Department of Health and Human Services) has been actively encouraged by proponents of DCD, including the Institute of Medicine [75,77]. The conflicts of interest inherent in this program aimed at increasing donation rates are obvious (Table 5) [97]. The so-called "team huddle" that allows early involvement of procurement coordinators with medical teams and critically ill patients bundles "what is in the patient's best interests (i.e. delivery of appropriate medical care) with the procurement coordinator's primary interest (i.e. securing consent to donate...) [[49]p1075]. Perhaps most telling is that a solution to perceived barriers to consent and conflicts of interest in obtaining consent is to have a trained representative of the organ procurement organization engage in the "impartial" donation discussion [75,97]. These trained requestors can then emphasize the "opportunity" to donate, that doing so "can save the lives of one or more people", and thereby "give back something to the community in return for what we have received from it through life", that donation "does not harm anyone", and it is "a way of passing on the gift of life to others" [98]. The "Spanish Model" of organ donation with high consent rates is based on: in-house intensive care/anesthesia physicians who are transplant coordinators and participate in treatment of the patient, and who have a basic low pay with incentive bonuses tied to organ retrieval success; comparisons between centers to foster competition; and less withdrawal of life support resulting in therapeutic ventilation and high proportions of brain deaths after brain injury [99-101].

**Table 5:** The Organ Donation Breakthrough Collaborative Best Practices, and Conflict of Interests.

Category	**Examples quoted from the published report **[97]	Page
Job Security	Hiring, supervision, and recognition are linked to performance [rates of consent].	v, 10, 30
	Accountability among hospital staff may be driven by hospital administration... Hold their staff accountable to performance.	v, 11-12
	Regularly track performance, and use data systems to track results at the staff member and organizational levels. Staff are held accountable.	v. 30
Healthy Competition	Reporting data by ICU fosters a healthy competition among units.	11
	Nurses reported that this sense of competition led to improvement in referrals and consents.	63
Call Early	Nurses automatically look at the white board to see if any patients look like potential donors.	viii, 44
	Conduct regular rounds in high potential ICUs... They are the most likely OPO personnel to identify potential donor cases early; they raise hospital staff awareness...	44,
	In house coordinators interacted with families as extensions of hospital nursing staff... OPO staff do not "hover" waiting for organs but do discretely monitor the patient's condition.	14, 56
	He is already thinking about organ donation upon the arrival of certain types of patients in the emergency room.	55
Goal is "yes"	Getting to an informed "yes" is paramount.	ix, 29, 57
Bordering on coercion	Assigns only those nurses [champions] to potential donor cases.	56, 58
	Importance of 'setting the stage' for consent well ahead of the declaration... becoming familiar with family dynamics and establishing a relationship with the key family decision-makers... bringing the family food and blankets... this type of 'surveillance' information was reported to be extremely useful in tailoring approaches to families for consent.	57, 58
	[Use the requestor] with the strongest connection or bond with the family... and has a history of achieving families' consent to donate.	58
	Presenting the donation request as a personal story, giving examples of transplant recipients.	61
	Proximity to transplant recipients as an opportunity to heighten the immediacy of need for organ donation.	68
Incentives	Often given incentives to perform well: compensation, bonuses, performance reviews... Financial incentives for achieving these targets [consents per year]...	11, 23
	Sessions at times 'when staff are hungry'... and bring more than enough food to serve all attendees.	42, 49
	Distributes pens, notepads, and mugs.	44
	Invites physicians, residents, and nurses to baseball games, hockey games, annual dinners and other outings to maintain buy-in, strengthen relationships, and recognize high performance.	49, 51
	Visit high-referring ICUs with dinner... sent the physician a box of his favorite cigars.	49
Business model	Strategically recruits high profile members of business and civic community to sit on the board of governors... Strategically appoints top officials from high donor potential hospital to its board... Strategically select influential, potentially pro-donation hospital personnel to serve on their boards... they do expect them to be champions for organ donation and accessible to the OPO for immediate as well as longer-term needs for facilitating organ donation.	20, 37, 45
	Orient operations towards outcomes rather than processes.	28
	If you secure doctors of high stature, it will facilitate mid-level doctor support.	47
	They serve as a 'committee of ears'...	47

Although most consensus statements recognize potential conflicts of interest, we believe they make misleading claims that these conflicts are dealt with and therefore not a concern.

## V Premortem Interventions, and the Principle of Double Effect

Premortem interventions are often used to theoretically improve the condition of donated organs in DCD, although there is no evidence that they improve outcomes [102]. These interventions include insertion of cannulas to allow rapid preservative solutions to be infused on declaration of death, administration of heparin to prevent clotting when circulation stops, and administration of phentolamine to improve perfusion to the organs during the process of dying (while still living). There are several problems with these practices, even with consent.

First, these interventions are, in theory, to benefit the organ recipient, not the donor. It is questionable whether the donor can consent to an intervention that cannot benefit him/her and has a significant risk of harm and of hastening or causing death. It has been argued that these interventions are unlikely to hasten or cause death [3,4]. We believe that this is inaccurate. If heparin was very safe, we would not carefully consider who we give therapeutic heparin to, and would not worry about life-threatening bleeding complications. If phentolamine was very safe, we would not worry about hypotension induced in patients it is given to. The fact is, these are potentially dangerous medications, with potentially life threatening complications. Bleeding, and hypotension, and anesthesia for inserting cannulas could each either make it more likely that death will ensue on withdrawal of life support, or that death will be hastened after withdrawal of life support. These complications can and do occur even in patients without high risk for their development. For example, in adults the risk of major bleeding associated with therapeutic heparin is up to 3%, is higher in the setting of ischemic stroke or after recent surgery or trauma, and still higher with higher doses of heparin [103]. In children, the risk of major bleeding with therapeutic heparin in PICU patients was 24% [104,105]. In DCD, the dose of heparin used is much higher than the therapeutic doses used in these studies. Moreover, if heparin prevents no-reflow phenomenon in brain, it may actually prolong the time needed for brain death to occur.

Second, we agree with others that "to summon the protection of the principle of double effect by claiming that since donation benefits the patient and family by complying with their wishes, use of these agents is permissible, is an extreme example of professional sophistry" [106]. In the principle of double effect as applied to heparin administration, four factors must be present: the action (giving heparin) must be intrinsically good (obtaining functional organs); the bad effect (death) may be foreseen but the agent must only intend the good effect; the bad effect must not be a means to the good effect; and the good effect must be proportional to (compensate for, or outweigh) the bad effect [107]. We question whether the bad effect (death) is not intended, whether the bad effect (death) is not a means to the good effect (obtaining functional organs), and whether the good effect (obtaining functional organs) is proportional to the bad effect (death). Weisbard wrote:

... the so-called 'foreseen but unintended' bad consequence in a double effect argument must be genuinely not desired... The presupposition of the conventional double effect argument is that the patient's death is the foreseen, but unintended and undesired, consequence of an effort to relieve pain... I find it simply impossible to take these assurances at face value in the context of a transaction whose entire purpose and reason for being is to bring about the death of the patient in a fashion that produces viable organs for transplantation... the protocol seeks to recharacterize a coherent, carefully worked out chain of events, leading inexorably to a foreseen, desired and planned result-death and utilization of organs for transplant- as a series of isolated links, each to be understood solely as directed to the patient's needs of the moment, each entirely disconnected from all surrounding context, including the very purpose of the exercise...[[43]p222].

## VI Straw-man arguments

We have found that it is common to respond to some of our concerns with so-called "straw-man" arguments. The Oxford dictionary of philosophy writes "to argue against a straw man is to interpret someone's position in an unfairly weak way, and so argue against a position that nobody holds, or is likely to hold" [108]. The arguments are against points that are not held by the opponent, and include: those opposed to DCD are simply against organ donation; and that we do not acknowledge that the family are often the ones who want to donate in DCD, the family deserves the opportunity to donate and have some good come of a terrible event, organ transplantation saves many lives, and thousands of people die every year on a transplant waiting list [109,110]. To clarify our position on these arguments: we are not against organ donation; the family that wants to donate in DCD is not truly informed when they consent to donation *after death*; the family does deserve the opportunity to donate in appropriate circumstances; organ transplantation saves many lives; and people do die on transplant waiting lists. Another claim is that surveys show the public is willing to accept DCD [4,111]. This is simply misleading, given that the public in these surveys is asked if they agree to organ donation *after death*, without explanation about the controversy in diagnosis of death [112,113]. When the controversy is revealed, surveys do not suggest strong support for the deception [112-114]. We do not believe these arguments have any bearing on whether the donor in current DCD protocols is actually dead. Further education on these straw-man arguments is not necessary. We agree with Zamperetti et al when they wrote "declaring that the patient's death has already taken place is morally questionable and scientifically untenable... the risk [is] of confusing genuine education and adequate awareness with manipulation of people's opinions..."[[115]p1674].

There are argument *against *DCD that we recognize also may be straw-man arguments. The process of DCD has been suggested to alter good end-of-life care. Some have called this "the forgotten donor" [116]. The recent Presidents Council was concerned that: families offered DCD may feel pressured to decide in favor, the process may interfere with good palliative care, the family's emotional needs and mourning may suffer ("considering that loved ones must be kept 'out of the surgeons' way' immediately after the patient's heart stops beating") [[51]p82], and rushing to make a death determination "could make the donor's death seem like a mere formality" [[51]p86]. We list this as a straw-man argument because it does not influence the debate about irreversibility of death, and it is unclear whether proponents of DCD would disagree with it. The recent cases of infant heart donation after 75 seconds of absent circulation demonstrate the extremes that DCD protocols have gone to [117]; nevertheless, many proponents of DCD have criticized this extreme time interval to declaration of death making it a position they do not hold [5]. The suggestion that heart transplant after DCD demonstrates the reversibility of cardiac death has been suggested by proponents of DCD as missing the point: it is circulation and not heart function that is to be permanently lost [5,10,118,119]. Some consider this sophistry (after all, the debate about autoresuscitation applies to heart function, and the normal cases rely on diagnosis of the loss of heart function), and are unconvinced (circulation is restored in the recipient, likely demonstrating the reversibility of absent circulation in the donor; saying loss of circulation was permanent because the heart was removed, and the heart was removed because loss of circulation was permanent is circular) [15,120-122]. We do not focus on this debate in this paper.

## VII The overwhelming consensus

The concerns with DCD should be debated on their merits without undue deference to prominent consensus statements of expert opinion. Nevertheless, we recognize that several respected groups have published consensus statements that have been said to affirm that DCD as currently done is ethically sound [1-5,75,123]. We do not believe these statements adequately address our concerns.

Consensus statements in general, and those on DCD in particular, are of low quality when assessed by evidence based medicine standards [124]. The main limitations are in the categories of stakeholder involvement, rigor of development, and editorial independence (Table 6) [125]. Most of the statements involve predominantly transplantation experts and lay public that are either transplant recipients or known to support transplantation, have not systematically reviewed the literature on autoresuscitation or Lazarus phenomenon, have been based on expert opinion, have had unclear panel selection and consensus building methods, and have been funded and organized by transplantation organizations. Many explicitly acknowledge that their objective was not to determine whether DCD complies with ethical norms or the dead donor rule [1,4,5,75,123].

**Table 6:** Consensus statements regarding donation after cardiocirculatory death from prominent medical groups, and some comments.

Consensus Group	Funding	Stated Goal	Examples of concerns
National Conference on DCD, 2006 [3]	Transplant organizations	"To address the increasing experience of DCD and to affirm the ethical propriety of transplanting organs from such donors...[and] to expand the practice of DCD in the continuum of quality end-of-life care."	1. "By new developments not previously reported, the conference resolved controversy regarding the period of circulatory cessation that determines death and allows administration of pre-recovery pharmacologic agents." These new developments were not described nor referenced. 2. Claim there are two different mutually exclusive types of death such that "the cardiopulmonary criterion may be used when the donor does not fulfill brain death criteria."
Interdisciplinary panel, 2010 [5]	Transplant organization	"To re-examine the standards for death determination and to analyze the new protocols' compliance with these standards."	1. Claim that death is "fundamentally a medical practice issue and not primarily a moral or ontological issue [[40]p1762]." 2. Claim that permanent cessation of circulation "is the meaning of 'irreversible' in the Uniform Determination of Death Act." Therefore, it "is ethically and legally appropriate to procure organs when permanent cessation of circulation has occurred but before irreversible cessation." Yet, the lead authors previously wrote that 'permanent' is not compatible with the intent of the UDDA [41,42].
Institute of Medicine, 1997 [1]	Transplant organization	"This report examines medical and ethical issues in recovering organs from NHBDs who do not meet the standard of brain death."	1. Accepted the premise "at the outset [that] recovery of organs from NHBDs should be considered a reasonable source of organs whose potential deserves serious exploration... It can be concluded, therefore, that the recovery of organs from NHBDs is an important, medically effective and ethically acceptable approach to closing the gap...[p45-46]" 2. The meaning or irreversible "must rest on expert medical opinion [p59]." 3. Ethical issues were presented to the IOM staff by "the chair of the committee that developed the Pittsburgh protocol for NHBDs in 1992 [p85]." The project heard presentations from those representing "medical and surgical transplantation, organ procurement, the bioethics of transplantation, donors, recipients, and the federal government [p45]."
Institute of Medicine, 2000 [123]	Transplant organization	"An effort designed to facilitate the adoption by all OPOs of protocols regarding NHBD."	1. Did not re-address the determination of death. Affirmed two different types of death: "the UDDA specifies the irreversible loss of all brain function *or *the irreversible cessation of cardiopulmonary function, not both [p24]."
Institute of Medicine, 2006 [75]	Transplant organization	"To study the issues involved in increasing the rates of organ donation."	1. Did not re-address the determination of death. Claimed that 'permanent' is "a reasonable interpretation of the concept of 'irreversibility' and is compatible with the probable intentions of the Commission that formulated the UDDA definition and with the UDDA's reference to 'accepted medical standards' [p172]." 2. Suggested uncontrolled DCD with reinstitution of CPR/ECMO, and supporting adopting the organ donation breakthrough collaborative, and suggested adopting "steps for preparation for such donation to the end of their [American Heart Association] standard resuscitation protocols [p185]." 3. A national workshop including "care providers, organ procurement professionals, and families who supported non-heart-beating donation [p2]."
SCCM Ethics Committee, 2001 [2]	Unclear	"To comment on the issues of timing of death."	1. Suggest a long observation time for certification of *all *deaths "flies in the face of both logic and the contemporary notion of death certification [p1872]." However, they did not discuss that in the context of DCD, and *unlike in the 'routine' death diagnosis*, following the dead donor rule requires knowing whether the patient is merely dying or already dead; diagnostic errors are not allowed, a retrospective diagnosis is not possible, and the long time of observation is required.. 2. "Did not achieve unanimity regarding the single 'best' observation period for asystole, apnea, and unresponsiveness."
Canadian Forum, 2006 [4]	Transplant organization	"To inform and guide health care professionals involved in developing programs for DCD... Discussion at the forum was restricted to optimal and safe practice in the field as it pertains to DCD."	1. Presentations were heart "by experts from international jurisdictions where DCD is currently practiced [pS2]." Adopted "a weaker interpretation" of irreversible, apparently simply echoing the IOM and SCCM reports. 2. Claim that "there has been speculation that a phenomenon known as autoresuscitation may exist." 3. Claimed that "based on animal studies and isolated human case reports electrical function of the brain ceases within 20s after circulatory arrest [pS8]," not acknowledging that this EEG activity reflects only the superficial cortical activity of cerebral hemispheres, not adequate to suggest brain death has occurred [58].

There is a large and growing list of authors who have raised many of our concerns with DCD, and who have concluded that DCD donors are not dead [13-16,46,48,49,106,115,116,126-132]. This list includes the recent President's Council: "In truth, there is reason to doubt that the cessation of circulatory and respiratory functions is irreversible, in the strict sense... To call the loss of functions irreversible, it must be the case that the functions could not possibly return, either on their own or with external help... If this [attempted resuscitation] were to occur, the patient would certainly not have been 'resurrected', but instead would have been (according to the cardiopulmonary standard of death) resuscitated, i.e., prevented from dying" [[51]p83]. They do not settle the issue, writing that, with more research, "assurance that the heart will not restart on its own within the relevant time frame, combined with an informed decision by the patient and family in favor of controlled DCD, may or may not be sufficient as a moral warrant for declaring death...[[51]p87]. They do suggest that the family should be informed of "the controversies about irreversibility" and that "there ought to be a broader public discussion and debate about the propriety of controlled DCD" [[51]p86].

## VIII Pediatric Considerations

The practice of DCD has been endorsed by the American Academy of Pediatrics, including the concerning suggestion for "timely referral...[that] may start in the emergency department with the admission of a critically injured child... Deliberation with the OPO should occur before or when...'withdrawal-of-care' or 'do-not-resuscitate' options are being discussed" [[133]p823]. Of further concern, it was later clarified that when referring to "deciding when human beings are dead... it is not the purpose of this policy statement to answer those questions, but to raise awareness of them" [134].

Concerns with DCD are increased in children. First, there is even less data regarding autoresuscitation and Lazarus phenomenon; only 6 children were included in the studies identified by the Institute of Medicine (Table 2) [19-21], and we are aware of only three published cases of (the early form of) Lazarus phenomenon in children [38,135]. This makes determination of "accepted medical standards" in declaring death problematic. Second, conflicts of interest are even more difficult to ignore in pediatric critical care, where units are multidisciplinary, and the same personnel care for potential donors and recipients [136]. Variability in end-of-life decisions among pediatric intensivists [87,137], and lack of validated prognostic systems for critically ill children increase the risk of premature determination of withdrawal decisions [138]. Third, it is unclear how to apply informed consent to the DCD setting in children.

In children (particularly under age 14 years), premortem decisions are made using the best interests standard by surrogate decision makers, usually the parents [139,140]. This differs from adults where decisions are often made by a substitute decision maker based on the previously expressed wishes of the patient (made when he/she was competent) [140]. For the never having been competent child the primary concern should be the best interest of the child, based on a complete and truthful assessment of benefits and burdens from the perspective of the child [139-141]. The obligation is not to society or the health care system; rather, it is to the child [140]. The best interests standard "does not fit well" with the process of DCD because there is no benefit to the child [2]. It is unclear how to justify the statement that "every family should be given the opportunity to allow their loved one to become an organ donor" [[133]p826]. The Society of Critical Care Medicine stated that "an altruistic model argues that organ donation will result in such great benefit to both the family of the deceased child and to the recipient families that the intervention is justified. Currently, there is broad support for organ donation following death in pediatric patients after appropriate informed consent" [[2]p1829]. However, they also write that "altruistic motives for donation cannot be presumed or inferred for pediatric patients" [[2]p1829]. Particularly in DCD, we are worried children may be used as a means to an end, since only others benefit from the donation. The child may experience anxiety, loneliness, and fear of abandonment/isolation due to lack of optimal palliative care as life support is withdrawn in the operating room [141]. The family may be deprived of the ability to perform death rituals, of holding the child as they die, and extended family/friends may be excluded from being with their loved one while living their final moments or hours [141]. The effects on the quality of death, and the subsequent grief of the family/friends are unclear.

These concerns are not merely theoretical. A recent review of pediatric DCD policies found variable and concerning practices (Table 7) [142]. Similar variability in policies has been found in adult DCD protocols [1,123]. The remarkable variation in DCD protocols, including the timing and techniques to determine absent circulation, suggest a lack of "accepted medical standards."

**Table 7:** Concerns with policies on donation after cardiocirculatory death in children's hospitals in the United States, Canada, and Puerto Rico [142].

Topic of concern	Examples	% of protocols
Death determination	Pulselessness can be determined by palpation alone (a highly inaccurate method [161,162].	14%
	No specification of method to determine pulselessness.	11%
	No specification of duration of absent circulation until organ harvest.	10%
	Fewer than 5 minutes of absent circulation until organ harvest.	10%
Conflicts of interest	Transplant personnel are precluded from declaring death.	88%
	Transplant personnel are excluded from premortem donor management.	51%
	Physicians caring for potential organ recipients are excluded from participating in premortem donor management or declaration of donor death.	32%
	If the family raises a question about organ donation, donation after cardiocirculatory death can be discussed with the family prior to a withdrawal of life support decision.	21%
Premortem interventions	Premortem interventions are prohibited.	3%
	Premortem heparin is used.	55%
	Premortem vasodilator(s) are used.	18%
	Premortem vessel cannulation is used.	36%
	Consent is required for premortem interventions.	75%
Palliative care of donor	Medication intended to hasten death is precluded.	44%
	Withdrawal of life support occurs only in the operating room.	54%
	Of those having withdrawal of life support in the operating room, the family is allowed to remain until death is declared.	48%
	The family is permitted to view the body after organ removal.	27%
Voluntariness of consent	The family can withdraw consent at any time.	16%

## IX The need for informed consent

That DCD violates the dead donor rule leads to important implications. If the dead donor rule is inviolable, then we must change the practice of organ donation to make it truly consistent with the dead donor rule, risking the lower quality of organs that would be donated. If the dead donor rule is not inviolable, then informed voluntary consent in terminally ill patients to violate the dead donor rule and allow organ donation as the proximate cause of death is required [6,7,126,127,143]. The only argument for maintaining the status quo would be to point out the good consequences that result, including saving lives by organ transplantation and maintaining trust in the medical/transplantation systems [5,8,9,144]. However, we believe that consequentialist calculations in defining death are irrelevant given that our concern is the actual state (death) of the patient. We seek to diagnose the univocal state of death, regardless of the consequences. As Nair-Collins has pointed out, "biological reality [biological death] is what it is, whether we like it or not... What the argument [from utility] advocates, however, is for the medical community to intentionally deceive the public about the biological reality of death" [[145]p681]. He goes on to say that "trust is at the foundation of medicine...[the argument] advocates doing something that is antithetical to the very existence of the institution of medicine..."[[145]p681]. Similarly, others point out that the most good/bad consequences can do "is give us a reason for keeping quiet about (or exaggerating) the real status of the condition. The bad consequences cannot stop a condition from being a disorder... it is not clear that that would justify anything other than a piece of large scale public dishonesty" [[146]p67].

We believe that truthful, complete, voluntary, fully informed consent to organ donation is required. This best respects patient autonomy [139,140,147]. Signed donor cards and donor registries do not indicate fully informed consent to organ donation; the information provided to the potential donor on organ procurement organizations websites is at best incomplete [148]. We agree with Browne that "if the aim is not just to maintain trust, but to do so by being trustworthy, deliberate deception that bypasses transparency and consent is forbidden... The real issue at stake is thus not what the IOM identifies, but whether trustworthiness is a value to be sought" [[14]p85].

A potential challenge to our call for fully informed consent could be the claim that organs are property, and organ donation is governed by gift law. It has been argued that organ donation would thus require only a donation intent, and not informed consent, as there are neither risks nor benefits to a *deceased *donor from donation, and the decision may occur completely outside the doctor-patient relationship (as in signing a donor card or onto a donor registry) [149-151]. This view is reflected in the current OPTN "proposal to update and clarify language in the DCD model elements" that has changed the wording of "consent" [implying informed consent] to "authorization" [152,153]. We believe this change is neither good policy nor an acceptable answer to our concerns, for several reasons. First, authorization of, and the intent of the "anatomical gift" is conditional on the death of the donor; if the donor is unaware that DCD violates the dead donor rule, it would be hard to argue that the gift was voluntarily authorized. By violating the dead donor rule, DCD would be a form of living donation, and donation of a vital organ a form of physician assisted death. For this reason, we believe that mere "authorization" would be inadequate, and that DCD should surely require fully informed consent when and if allowed at all [154,155]. Second, authorization of the "anatomical gift" outside of the doctor-patient relationship assumes that the diagnosis of death is made objectively by doctors, without (even unconscious) bias in the decision to withdraw life-support or determine death. Of note, the OPTN proposal strikes any reference to the decision to withdraw life support being made before evaluating a patient as a DCD candidate, or of needing confirmatory tests of absent circulation (such as arterial line monitoring) [152]. Third, *pre*mortem interventions are not done after death, and have real harms and benefits that require informed consideration by potential donors. In addition, the end-of-life (prior to death) care provided to the donor is altered and should require informed consent [116]. Fourth, some argue that it is not clear that DCD donors are incapable of an experience of pain or suffering [7,154,156], particularly if circulation is re-established with CPR or ECMO. Whether raw affective experience from brainstem and subcortical structures [157] is possible at 2-5 minutes after absent circulation is unknown.

The United States Center for Medicare and Medicaid Services has held that "... there must be a minimum standard to assure that when families provide consent, they are providing informed consent... potential donor families receive the information they need to make an informed decision about donation..." [158]. A large group of pediatric intensive care clinicians, in responding to our call for a moratorium on DCD, claimed that a moratorium would deprive parents of the "ability to make a truly 'informed' decision about what we should hold sacred, how one chooses to die" [159]. In view of these statements by proponents of DCD, it is surprising that the OPTN proposal seeks to remove an informed consent requirement for DCD. We agree with others that the dead donor rule that is said to justify the gift law interpretation of authorization for organ donation after death hides the normative nature of the donation decision, and "disguises moral judgments by pseudo-objective claims [about death]" [7].

## X Conclusions: a call for a moratorium on DCD pending fully informed consent

We have argued that DCD donors are not dead, and therefore that organ donation during DCD violates the dead donor rule. Our concerns with DCD include the following: irreversibility of absent circulation has not occurred and the many attempts to claim it has all fail; conflicts of interest at all steps in the DCD process are simply unavoidable; premortem interventions to preserve organ utility are not justifiable; and consensus statements by respected medical groups do not change these arguments. The truth, we believe, is that honesty requires that we face these problems instead of avoiding them. Until the concerns we describe are seriously considered, full public disclosure occurs, and fully informed consent is obtained from donors, there should be a moratorium on the practice of DCD. We believe that DCD is not ethically allowable because it abandons the dead donor rule, has unavoidable conflicts of interests, and implements premortem interventions which can hasten death. These important points have not been, but need to be fully disclosed to the public and incorporated into fully informed consent. These are tall orders, and require open public debate. Until this debate occurs, we call for a moratorium on the practice of DCD.

## Competing interests

The authors declare that they have no competing interests.

## Authors' contributions

ARJ drafted the first version of the manuscript. All authors made substantial contributions to conception and design of the manuscript, revising the manuscript critically for important intellectual content, and have read and approved the final manuscript. Each author participated sufficiently to take public responsibility for the content. There was no funding for this manuscript.
